# Optimization of Desalting Conditions for the Green Seaweed *Codium fragile* for Use as a Functional Food with Hypnotic Effects

**DOI:** 10.3390/foods13203287

**Published:** 2024-10-16

**Authors:** Sohong Park, Duhyeon Kim, Seonghui Kim, Gibeom Choi, Hodeung Yoo, Serim Park, Suengmok Cho

**Affiliations:** Department of Food Science and Technology, Institute of Food Science, Pukyong National University, Busan 48513, Republic of Korea; sohong8401@gmail.com (S.P.); dueatnow@gmail.com (D.K.); shkim.pknu@gmail.com (S.K.); gbcpknu@gmail.com (G.C.); hdyoo.pknu@gmail.com (H.Y.); gsh05018@gmail.com (S.P.)

**Keywords:** *Codium fragile*, desalination, green seaweed, optimization, functional food

## Abstract

*Codium fragile* (CF) contains various bioactive compounds, but its high salt content (39.8%) makes its use as a functional food challenging. Here, we aimed to optimize the desalination process and verify changes in functionality based on variations in salt and total phenolic contents. To optimize the CF immersion conditions for the lowest salt content and monitor the total phenolic content, a response surface methodology was used. The optimal immersion conditions were as follows: *X*_1_ (immersion temperature) = 42.8 °C; *X*_2_ (immersion time) = 1.0 h. An inverse correlation was noted between salt content and total phenolic content. Among the post-desalination processes, desalination with centrifugal dehydration (CD) significantly reduced salt content. CD ethanol extract (CD-E) induced the longest sleep duration in the pentobarbital-induced sleep test in ethanol extracts. Moreover, 1000 mg/kg CD-E had a significant effect on non-rapid eye movement sleep but did not affect delta activity. These findings highlight the potential of industrializing CF as a functional food through desalination and its promise as a natural aid for sleep promotion.

## 1. Introduction

A species of green seaweed, *Codium fragile* (CF), is frequently observed in the coastal areas of East Asia, Northern Europe, and Oceania [1]. Oriental medicine has a long history of use for the treatment of internal diseases [2]. CF contains a variety of bioactive compounds with anti-cancer, anti-obesity, anti-inflammatory, and anti-oxidant properties [3,4,5,6]. Bioactive compounds in seaweeds are attracting interest as potential ingredients for functional foods, due to their safety and bioactive efficacy, in addition to showing promise in sleep health [7,8]. For instance, the sleep-promoting effects of brown seaweeds, specifically *Ecklonia cava*, have been demonstrated [9]. Although green seaweeds such as CF could serve as natural sources with hypnotic effects, they have not yet been investigated comprehensively.

CF is a high-salt seaweed typically containing 8–13 g sodium per 100 g (dry basis) [10,11]. Extraction with water or ethanol concentrates the salt, increasing the salt content in the CF extracts. The high salt content of CF and its extracts significantly limits its use as a functional food for health purposes.

Persistent intake of salt-rich foods has been associated with health complications, including hypertension and chronic kidney disease [12,13]. Several countries, including South Africa and Saudi Arabia, have established regulations regarding the maximum permissible sodium levels in certain food categories [14,15]. A trend toward low-salt foods is growing because of regulatory measures for reducing salt intake and rising consumer preference for healthier dietary options [16]. Therefore, it is crucial to identify an efficient desalination method that uses CF as a functional ingredient.

In this study, we aimed to investigate efficient desalting conditions for CF using distilled water immersion and a post-desalination process. The immersion conditions were optimized using response surface methodology (RSM). Changes in the phenolic content and hypnotic effects according to the degree of desalination were measured to evaluate the potential of CF as a functional food.

## 2. Materials and Methods

### 2.1. Materials

CF was harvested and dried on Wando Island, Korea. Pentobarbital was purchased from Hanlim Pharmaceutical. Co., Ltd. (Seoul, Republic of Korea). The Folin–Ciocalteu reagent was purchased from Junsei Chemical Co., Ltd. (Tokyo, Japan). Phloroglucinol and doxepin hydrochloride (DH) were purchased from Sigma Aldrich (St. Louis, MO, USA). Sodium carbonate was purchased from Samchun Chemical Co. Ltd. (Seoul, Republic of Korea). All the chemicals and reagents used in this study were of analytical grade.

### 2.2. Desalination

#### 2.2.1. Immersion

Dried CF was immersed in distilled water at a ratio of 20:1 (*w*/*v*) under each RSM condition in a shaking incubator (DS-210SF, Daewon Science, Inc., Bucheon, Republic of Korea).

#### 2.2.2. Post-Desalination Processes

All the post-desalination samples were immersed in distilled water for 1 h at 20 °C under optimal immersion conditions. Desalination with sonication was carried out using a sonicator (Powersonic 620, Hwa Shin Technology Co., Seoul, Republic of Korea) operating at 40 kHz and 700 W during immersion [17,18]. Desalination with a vacuum pulse was executed according to the methodology described previously [19], involving a cyclic transition between a vacuum set at 100 hPa and atmospheric pressure conditions at 15 min intervals during immersion. Desalination by centrifugal dehydration was performed using a spin extractor (FD-08BL, Hanil Electric, Seoul, Republic of Korea) at 1600 rpm for 10 min following immersion.

### 2.3. Design and Statistical Analysis of Experiments

A central composite design (CCD) was used to optimize the immersion conditions. The CCD matrix comprised 2^2^ factorial points, 2^2^ axial points (α = 1.414), and 3 replicates of the center points. The independent variables were the immersion temperature (*X*_1_, °C) and immersion time (*X*_2_, h). The coded and uncoded experimental values for the range of the independent variables and center points are listed in Table 1. These ranges were established based on the results of the initial experiments. The dependent variables were salt content (*Y*_1_, %) and total phenolic content (TPC) (*Y*_2_, milligrams of phloroglucinol equivalents per gram). Experimental trials were randomly assigned to reduce the impact of unforeseen statistical errors across the 11 experimental groups. The RSM in Minitab statistical software (Version 19, Minitab Inc., State College, PA, USA) was used to analyze the experimental data, resulting in the derivation of Equation (1) for the generalized quadratic polynomial regression model, as shown below:

(1) $$Y=\beta_{0}+\overset{2}{\underset{i=1}{\sum }}\beta_{i}X_{i}+\overset{2}{\underset{i=1}{\sum }}\beta_{ii}X_{i}^{2}+\overset{1}{\underset{i=1}{\sum }}\overset{2}{\underset{j=i+1}{\sum }}\beta_{ij}X_{i}X_{j}$$

where Y represents the predicted dependent variable, $\beta_{0}$ is a constant, and $\beta_{i}$, $\beta_{ii}$, and $\beta_{ij}$ represent linear, quadratic, and interaction regression coefficients, respectively. $X_{i}$ and $X_{j}$ are coded as independent variables. For the CF desalination, the optimization variables considered were the dependent variables, that is, *Y*_1_ and *Y*_2_. Response optimization was performed by specifying the target values for minimizing *Y*_1_ and maximizing *Y*_2_ using the Minitab software. Maple software (Maple 7; Waterloo Maple Inc., Waterloo, ON, Canada) generated three-dimensional response surface plots using the fitted model equations.

### 2.4. Powder

To prepare samples for accurate measurements of salt content and TPC, both dried and desalinated CF were processed into powders. The desalinated CF was dried at 50 °C for 24 h in an oven (WFO-700; Sunil Eyela, Seongnam, Republic of Korea). Dried samples were turned into 0.5 mm sized powder using a mixer (HC-BL5100, HC Company, Gimhae, Republic of Korea) and sieve (Daihan Scientific Co., Seoul, Republic of Korea).

### 2.5. Extract

A methanol extraction method was used to assess TPC in both dried and desalinated CF. The powder was subjected to extraction using 80% methanol at a ratio of 10:1 at 20 °C for 24 h in a shaking incubator according to the procedure outlined in [9]. The extracts were centrifuged at 4000 rpm for 20 min. The extract supernatants were concentrated and dried in an oven at 40 °C. Ethanol extracts were prepared to evaluate their hypnotic effects. Dried CF, a sample of optimal immersion conditions, and a desalination sample that underwent centrifugal dehydration were extracted using 70% ethanol at a ratio of 10:1 for 14 h in a shaking incubator at 60 °C. The ethanol extract was filtered using filter paper (5C 110 mm; Advantech, Tokyo, Japan). The filtrate was concentrated under a vacuum at 50 °C and lyophilized.

### 2.6. Determination of Salt Content

Preprocessing was necessary because of the dark color of the powder or ethanol extracts [20]. Approximately 2–3 g samples were heated in an electric furnace at 550–600 °C until their color turned light gray. A modified version of the Mohr method [21] was used to determine the salt content. After heating, the ash was solubilized to 500 mL with distilled water. Further, 10 mL of the solution was added to a triangular flask along with two to three drops of 10% (*w*/*v*) potassium chromate solution. The titration procedure was conducted using a burette filled with a 0.02 N solution of silver nitrate. Salt content was calculated using Equation (2), as shown below:

(2) $$\mathrm{Salt}\;\mathrm{content}\;(\%)=\frac{V_{AgNO_{3}\;}\times F\times 0.00117\times V_{T}}{W_{sample}\times V_{sample}}\times 100$$

where $V_{AgNO_{3}}$ is the volume of 0.02 N AgNO_3_ consumed during titration, F is a factor of 0.02 N AgNO_3_, $V_{T}$ is the total volume of the ash solution, $W_{sample}$ is the mass of the sample used for ashing, and $V_{sample}$ is the volume of the ash solution used during titration.

### 2.7. Determination of TPC

TPC was assessed using the Folin–Ciocalteu method [22]. The methanol and ethanol extracts were solubilized in methanol. The Folin–Ciocalteu reagent (0.5 mL) was added to 8 mL of distilled water and 0.5 mL of sample solution. After 3 min, 1 mL of a 20% (*w*/*v*) sodium carbonate solution was added. The mixture was incubated in the dark at an ambient temperature for 1 h. The sample was placed in a 96-well plate and the absorbance was measured at 765 nm using a spectrophotometer. TPC was quantified in milligrams of phloroglucinol equivalents per gram of sample (mg PGE/g, dry basis).

### 2.8. Energy-Dispersive X-Ray Spectrometer (EDS) Mapping of Sodium Ions on the Surface and Cross-Section

Dried CF and freeze-dried desalinated CF were prepared to confirm the surface and cross-sectional sodium distribution. All the samples were coated with platinum. Field-emission scanning electron microscopy (FE-SEM; MIRA 3, TESCAN, Brno, Czech Republic) was performed using an EDS (X-Max N, Oxford Instruments, Abingdon, UK) at an accelerating voltage of 10 kV and a magnification of 3500×.

### 2.9. Animals

Male Institute of Cancer Research (ICR, 20–25 g) mice and C57BL/6N mice (25–28 g) were purchased from Koatech Animal, Inc. (Pyeongtaek, Republic of Korea). The laboratory where the animals were kept was regulated for humidity and maintained at a temperature of 23 ± 0.5 °C, following a half-day light cycle controlled automatically, with lights turned on at 9:00 AM. Water and food were provided ad libitum. The guidelines were established by the Institutional Animal Care and Use Committee of Pukyong National University (Permit no. PKNUIACUC-2023-40, no. PKNUIACUC-2023-07) and were strictly followed to reduce suffering and the number of animals used.

### 2.10. Pentobarbital-Induced Sleep Test

The mice fasted for 24 h before the initiation of the experiments, which were conducted between 1:00 p.m. and 5:00 p.m. Intraperitoneal injection (i.p.) of pentobarbital (45 mg/kg) was administered to ICR mice 45 min after oral injection (p.o.) of the test samples. The ethanol extracts were solubilized in a sterile saline solution containing 5% (*v*/*v*) Tween 80. The mice treated with pentobarbital were housed individually in cages, and an observer, unaware of the sample groups, measured sleep latency and duration. Sleep duration was measured as the time from the onset of loss of the righting reflex to recovery.

### 2.11. Analysis of Sleep Architecture

The polysomnographic recordings were performed following a previous study [23]. Following an incision in the subcutaneous connective tissue of the head, the mice were fitted with a head mount (#8201; Pinnacle Technology, Lawrence, KS, USA) to the bregma of the skull. A head mount was used for electroencephalography (EEG) and electromyography (EMG). Four electrode screws were implanted, and the dual wires of the head mount were placed into the nuchal muscles on both sides. Dental cement was affixed to secure the device, and the mice were allowed 1 week for recovery.

A PAL-8200 data acquisition system (Pinnacle Technology, Lawrence, KS, USA) was used after oral administration of the sample, and EEG and EMG activities were continuously monitored for 24 h (starting at 5:00 p.m.). The EEG and EMG signals were amplified 100 times and low-pass-filtered at 25 Hz and 100 Hz, respectively. The signals were set to maintain a sampling frequency of 200 Hz. The control group comprised identical mice whose sleep–wake cycles were observed over a 24 h period, which included the collection of baseline data and experiments. The sleep stage, wakefulness (Wake), rapid eye movement sleep (REMS), and non-rapid eye movement sleep (NREMS) were classified using SleepSign Version 3 (Kissei Comtec, Nagano, Japan). REMS was categorized as theta activity ranging from 6 to 10 Hz, whereas NREMS was characterized by delta activity within the 0.65–4 Hz range. The onset time was defined as the moment when the first consecutive NREMS lasted for a minimum of 2 min following the administration of the sample. The percentage of delta power during the NREMS was calculated. Rapid improvements in the Wake, NREMS, and REMS were characterized by periods lasting longer than 10 consecutive seconds. Sleep structure analysis was performed using the fast Fourier transform (FFT) algorithm.

### 2.12. Statistical Analysis

Data are expressed as the mean ± standard error of the mean (SEM). Multiple comparisons were conducted using one-way analysis of variance (ANOVA) followed by Dunnett’s test. Comparisons between the two groups were made using unpaired and paired Student’s *t*-tests. Statistical analyses were performed using Prism version 10.2 (GraphPad Software Inc., San Diego, CA, USA).

## 3. Results

### 3.1. Hypnotic Effect of CF Ethanol Extract

**Figure 1:**
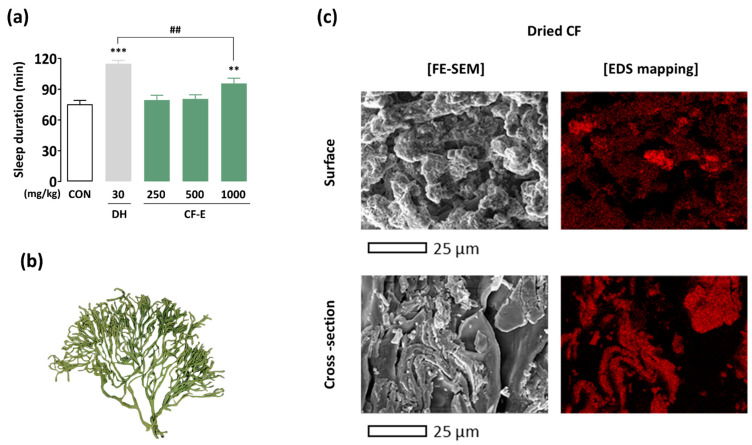
Hypnotic effect of CF-E and CF characteristics. (**a**) Influences of CF-E and DH on sleep duration in ICR mice. (**b**) Textural image of CF. (**c**) FE-SEM EDS images of the surface and cross-section of dried CF. The data are presented as the mean ± SEM (n = 7–8). ** *p* < 0.01 and *** *p* < 0.001: significantly different from the control group (Dunnett’s test). ## *p* < 0.01: significantly different between the two groups (unpaired Student’s *t*-test). CON: control; DH: doxepin hydrochloride; CF: *Codium fragile*; CF-E: *Codium fragile* ethanol extract; SEM: standard error of the mean.

To assess the potential of CF as a functional food, the hypnotic effect of CF ethanol extract (CF-E) was evaluated using the pentobarbital-induced sleep test in ICR mice (Figure 1a). DH, a widely recognized drug used for insomnia treatment, was used as a positive control. Compared with the control (CON) group (75.4 ± 3.7 min), DH (30 mg/kg) was observed to significantly prolong the sleep duration to 114.9 ± 3.2 min (*p* < 0.001). In varying doses (250–1000 mg/kg), the CF-E at 1000 mg/kg potentiated the sleep duration to 95.6 ± 5.2 min (*p* < 0.01). However, CF-E exhibited a statistically significant difference compared with DH (*p* < 0.01).

### 3.2. Texture and Sodium Ion Distribution of the Dried CF

The salt content of the dried CF was 39.8%; however, despite rinsing under running water, it remained at 18.8%. To understand the reason for the high salt residue, the textural image and sodium ion distribution of the dried CF were analyzed using FE-SEM and EDS mapping (Figure 1b,c). The CF exhibited a fluffy texture with spongy thali (Figure 1b), and the cross-section showed a higher concentration of sodium ions than the surface (Figure 1c). These results indicated that rinsing was ineffective for salt removal.

### 3.3. Optimization of Immersion Conditions of CF

#### 3.3.1. Evaluation of the Fitted Models Through Diagnostic Checking

The immersion conditions and results for *Y*_1_ (salt content) and *Y*_2_ (TPC) based on the CCD are listed in Table 2. To explore the correlation between the independent and dependent variables, it is crucial to formulate a second-order regression equation. The results for the linear terms (*X*_1_, *X*_2_), quadratic terms (*X*_1_*X*_1_, *X*_2_*X*_2_), and interaction terms (*X*_1_*X*_2_) of the three dependent variables are presented in Table 3. The constant coefficients for all dependent variables and *X*_1_ representing *Y*_1_ (salt content) and *Y*_2_ (TPC) were significant (*p* < 0.01). For *Y*_1_ (salt content), *X*_2_, *X*_1_*X*_1_, and *X*_1_*X*_2_ were significant (*p* < 0.05, and *p* < 0.01, respectively). Meanwhile, *X*_2_ and *X*_1_*X*_1_ of *Y*_2_ (TPC) were significant (*p* < 0.01 and *p* < 0.05, respectively). The positive coefficients in the *Y*_1_ model and the negative coefficients in the *Y*_2_ model indicate that as the values of *X*_1_, *X*_2_, and *X*_1_*X*_1_ increase, *Y*_1_ increases, whereas *Y*_2_ decreases. The R^2^ values for *Y*_1_ and *Y*_2_ are 0.965 and 0.936, respectively (Table 4).

#### 3.3.2. Analysis of Variance

The association between the independent and dependent variables in the response surface model equation was examined using ANOVA (Table 5). The linear, square, and interaction terms of the dependent variable *Y*_1_ (salt content) and the linear terms of the dependent variable *Y*_2_ (TPC) were statistically significant (*p* < 0.01). The squared terms of the dependent variable, *Y*_2_ (TPC), were also significant (*p* < 0.05). The lack-of-fit test model is considered a response surface model that fits the available data when the *p*-value exceeds 0.05 [24]. The adequacy of all dependent variables reached statistical significance (*p* > 0.05).

#### 3.3.3. Response Surface Plots and the Effect of Factors

**Figure 2:**
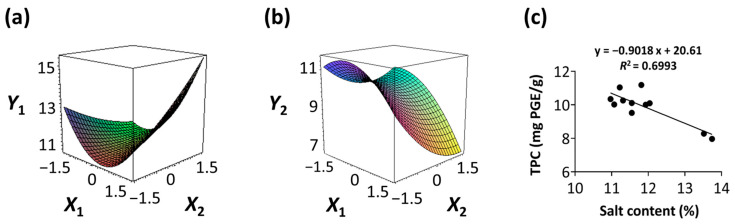
Three-dimensional response surface plots for (**a**) salt content (%) and (**b**) TPC (mg PGE/g). (**c**) Correlation between salt content and TPC in a central composite design matrix. TPC, total phenolic content; *X*_1_, immersion temperature (°C); *X*_2_, immersion time (h).

The effects of the immersion temperature (*X*_1_) and time (*X*_2_) on the salt content (*Y*_1_) and TPC (*Y*_2_) are depicted in a three-dimensional graph (Figure 2a,b). The salt content (*Y*_1_) demonstrated the lowest value at an immersion temperature (*X*_1_) of 42.8 °C (+0.199). Meanwhile, the immersion time (*X*_2_) reached its minimum at 1 h (−1.414) (Figure 2a). The peak value of TPC (*Y*_2_) was observed at an immersion temperature (*X*_1_) of 32.1 °C (−0.557) and decreased toward 60 °C (+1.414) (Figure 2b). As the immersion time (*X*_2_) increased from 1 h (−1.414) to 12 h (+1.414), a decreasing trend in TPC was observed. Phenolic compounds are the main components responsible for the hypnotic effects in seaweeds [9,25]. Therefore, the TPC and salt content were simultaneously monitored to investigate the potential correlation between the variance in the dependent variables (Figure 2c). An inverse correlation was observed between the salt content (*Y*_1_) and TPC (*Y*_2_), with an *R^2^* value of 0.6993. This implies that RSM can decrease salt in CF while also improving the TPC.

#### 3.3.4. Optimal Immersion Conditions and Verification

The optimal immersion conditions (OICs) were determined by considering the salt content (*Y*_1_) and TPC (*Y*_2_). In Table 6, the independent variables of the OICs are presented as coded and actual values. For meeting the criteria of minimizing salt content (*Y*_1_) and maximizing TPC (*Y*_2_) simultaneously, immersion temperature (*X*_1_) and immersion time (*X*_2_) were found to be +0.199 (42.8 °C) and −1.414 (1.0 h), respectively. To validate the model, the predicted values were compared with the actual values of the dependent variables [26]. The predicted values for the salt content (*Y*_1_) and TPC (*Y*_2_) of OICs were 11.0% and 11.2 mg PGE/g, respectively (Table 7). Under the OICs, the measured salt content (*Y*_1_) was found to be 10.9 ± 0.1%, while the TPC (*Y*_2_) was determined to be 11.2 ± 0.5 mg PGE/g. The actual values closely matched the statistically predicted values.

### 3.4. Post-Desalination Processes

Despite the reduction in salt content to 10.9% (Table 7) achieved through OICs, this level was still deemed excessive. Therefore, post-desalination processes were conducted (Table 8). The salt content of desalination with sonication (SN, 1.5%), vacuum pulse (VP, 1.4%), and centrifugal dehydration (CD, 0.7%) was significantly lower (*p* < 0.001) than that of OICs. The TPCs of SN, VP, and CD were also statistically higher (*p* < 0.001, respectively) than that of OICs, with values of 22.3, 22.6, and 28.5 mg PGE/g, respectively.

**Figure 3:**
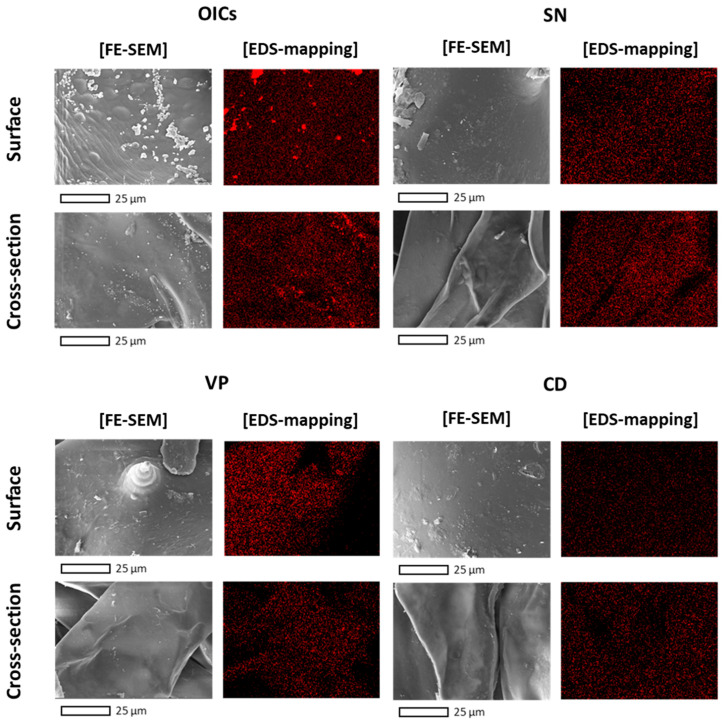
FE-SEM EDS images of surface and cross-section of OICs, SN, VP, and CD. OICs, optimal immersion conditions; SN, sonication; VP, vacuum pulse; CD, centrifugal dehydration.

To examine the alteration in the distribution of sodium ions throughout the desalination process, the OICs and post-desalination samples were analyzed (Figure 3). The Na distribution exhibited a pattern that matched the salt content of each sample. CD showed the most significant removal of sodium compared with OICs, both internally and externally. To examine changes in salt content after ethanol extraction, CF-E, OICs ethanol extract (OICs-E), and CD ethanol extract (CD-E) were prepared. CD-E exhibited a significantly lower salt content (17.2%) than CF-E (70.7%) and OICs-E (60.6%) (Table 9). The yield of CD-E (2.1%) was attributed to a reduction in salt content. The TPC was inversely related to salt content, with CD-E showing the highest TPC at 21.9 mg PGE/g.

### 3.5. Pentobarbital-Induced Sleep Test

**Figure 4:**
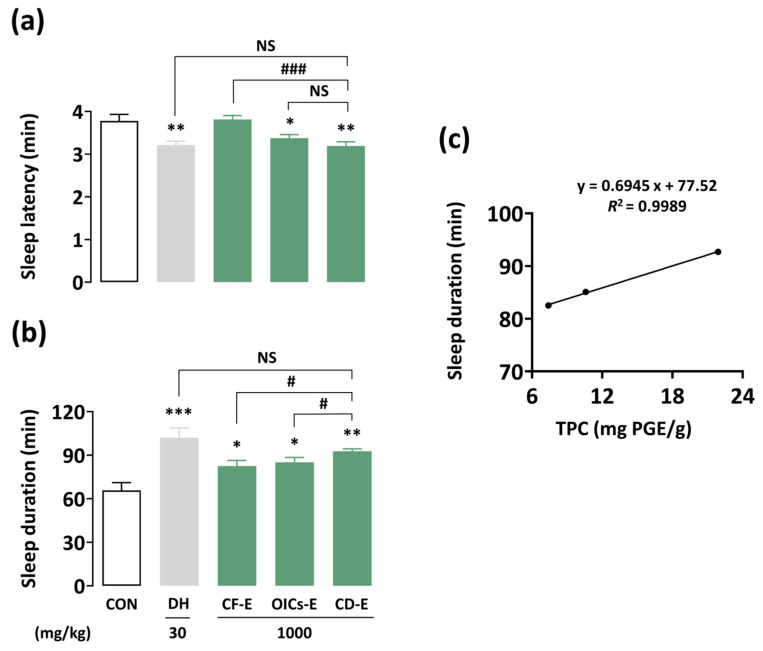
Influences of CF-E, OICs-E, CD-E, and DH on (**a**) sleep latency and (**b**) duration in ICR mice. (**c**) Correlation between TPC and sleep duration in ethanol extracts. The data are presented as the mean ± SEM (n = 7–8). * *p* < 0.05, ** *p* < 0.01 and *** *p* < 0.001: significant difference as compared with the control group (Dunnett’s test). # *p* < 0.05 and ### *p* < 0.001: a significant difference between the two groups (unpaired Student’s *t*-test). CON, control; DH, doxepin hydrochloride; CF-E, *Codium fragile* ethanol extract; OICs-E, optimal immersion conditions ethanol extract; CD-E, centrifugal dehydration ethanol extract; SEM, standard error of the mean; NS, not significant; TPC, total phenolic content.

The hypnotic effects of CF-E, OICs-E, and CD-E were assessed to monitor changes in functionality according to the degree of desalination (Figure 4a,b). All the samples effectively improved sleep duration. The sleep latency and duration of CD-E showed significant differences compared with those of the CON group at 3.2 ± 0.1 min and 92.7 ± 1.6 min (*p* < 0.01, respectively), but were not significant compared to the positive control, DH. CD-E exhibited strong effects compared with CF-E and OICs-E in terms of sleep duration (*p* < 0.05) (Figure 4b). Additionally, a potential correlation between the TPC and hypnotic effects of the ethanol extracts was observed (Figure 4c). The *R*^2^ 0.9989 suggests a strong relationship between the variables. Therefore, these results indicate that removing the high salt content from CF increases the TPC and produces an extract with enhanced functionality.

### 3.6. Analysis of Sleep Architecture

#### 3.6.1. Impact of CD-E on Sleep Patterns and Temporal Course Changes

**Figure 5:**
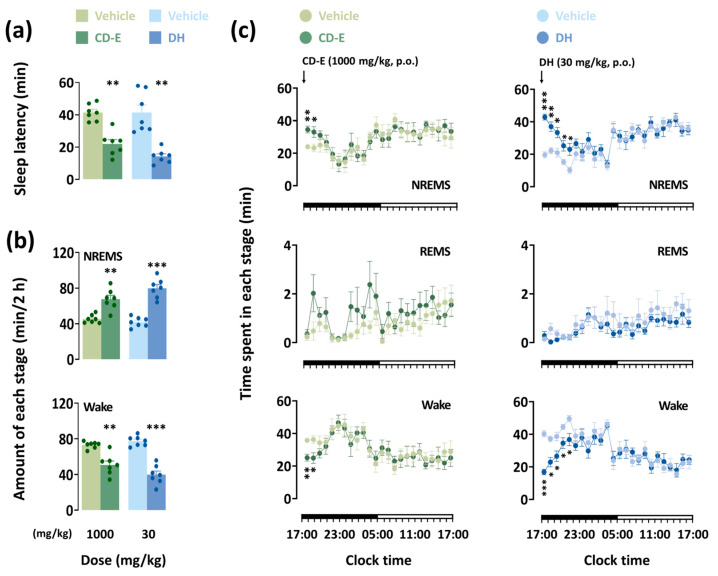
Effects of CD-E and DH on sleep patterns in mice. (**a**) Influences of CD-E and DH on sleep latency in ICR mice. (**b**) Influences of CD-E and DH on NREMS and Wake amounts during the 2 h period. (**c**) Influences of CD-E and DH on temporal course changes in amounts. Light and deep color circles indicate the baseline (vehicle) and experimental sample (CD-E or DH). The data are represented by the hourly mean ± SEM (n = 7–8). * *p* < 0.05, ** *p* < 0.01 and *** *p* < 0.001: significantly different from the vehicle (paired Student’s *t*-test). DH, doxepin hydrochloride; CD-E, centrifugal dehydration ethanol extract; NREMS, non-rapid eye movement sleep; REMS, rapid eye movement sleep; Wake, wakefulness; SEM, standard error of the mean.

To gain a deeper insight into the sleep-inducing effect of CD-E, we analyzed sleep architecture based on EEG and EMG recordings. In polysomnographic recordings, the oral administration of CD-E and DH showed a significant difference compared with the vehicle (Figure 5a,b). Sleep onset values were recorded as 21.9 ± 2.7 and 14.2 ± 1.6 min in treatments with CD-E (1000 mg/kg) and DH (30 mg/kg), respectively (Figure 5a). The amounts of NREMS and Wake were measured during the initial 2 h following the administration of CD-E or DH (Figure 5b). Oral administration of 1000 mg/kg CD-E was significantly enhanced by 1.5 times in NREMS (*p* < 0.01). CD-E decreased Wake by 0.7 times (*p* < 0.01). DH led to a significant increase of 1.9 times in the NREMS (*p* < 0.001). Wake was also significantly reduced by 0.5 times after administration of DH (*p* < 0.001).

CD-E treatment significantly increased the amount of NREMS within the first 2 h following injection by 1.4 times each (*p* < 0.01 and *p* < 0.05, respectively) (Figure 5c). This increase in NREMS coincided with a decrease in Wake. Compared with the vehicle, the hypnotic effects of DH persisted for 5 h after administration. Neither CD-E nor DH significantly differed in the number of REMS during 24 h after administration.

#### 3.6.2. Impact of CD-E on Sleep–Wake Episodes and Delta Activity

**Figure 6:**
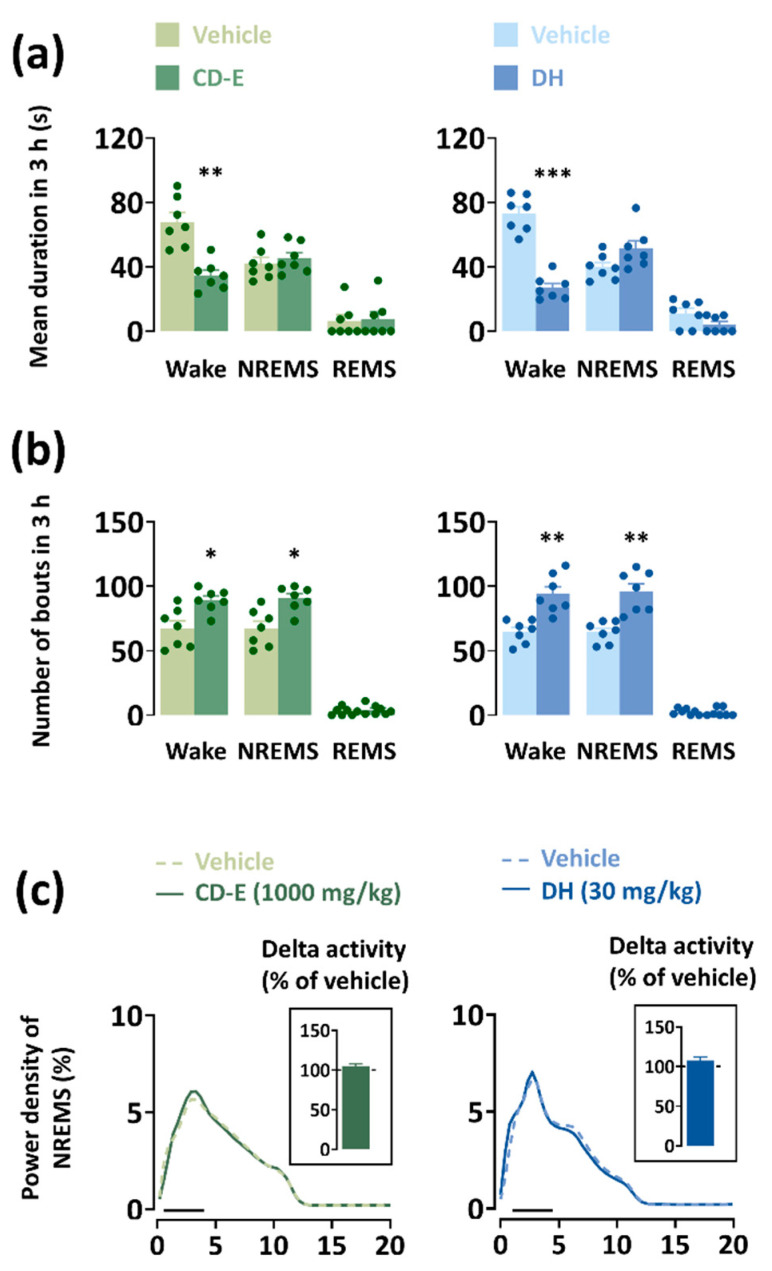
Influences of CD-E and DH on sleep–wake episodes in mice during the 2 h period after injection. (**a**) Mean duration and (**b**) total number of Wake, NREMS, and REMS bouts in 2 h after the administration of CD-E (1000 mg/kg) and DH (30 mg/kg). (**c**) EEG power density curves of NREMS caused by CD-E and DH. The solid bar (─) represents the range of the delta wave (0.5–4 Hz). Each value represents the mean ± SEM (n = 7–8). * *p* < 0.05, ** *p* < 0.01 and *** *p* < 0.001: significantly different from the vehicle (paired Student’s *t*-test). DH, doxepin hydrochloride; CD-E, centrifugal dehydration ethanol extract; NREMS, non-rapid eye movement sleep; REMS, rapid eye movement sleep; Wake, wakefulness; SEM, standard error of the mean.

Alterations in the mean duration and features of sleep–wake episodes caused by DH and CD-E were analyzed (Figure 6). CD-E and DH showed a reduction (CD-E: 49%, *p* < 0.01; DH: 63%, *p* < 0.001) in the mean duration of waking without changing NREMS and REMS (Figure 6a). CD-E augmented Wake and NREMS bouts by 1.3 times each (*p* < 0.05, respectively) (Figure 6b). To evaluate sleep depth, delta activity was computed in C57BL/6N mice during NREMS (Figure 6c). CD-E and DH did not affect the power density of the EEG or delta activity.

## 4. Discussion

CF, which has a high salt content of 39.8%, is difficult to use industrially without removing excess salt. The siphon structure of CF [27,28] led to significant accumulation of salt in the cross-section, as confirmed by EDS mapping (Figure 1c). In the present study, the salt content was reduced to 0.7% by immersion and post-desalination.

As water immersion is one of the most commonly used desalination methods [29,30], desalination processes in the present study were conducted through immersion. Temperature and immersion time are key factors influencing the desalination process [31]. Therefore, immersion temperature and time were the independent variables and RSM was conducted to determine the OICs for removing salt from the CF (Table 2). The increase in salt content with longer immersion times (Figure 2a) was likely due to an increase in the concentration of the solution by salt leaching from the CF. This likely caused osmotic dehydration. Therefore, the surface of the CF lost moisture and reabsorbed the solutes. Similar findings have been reported for the osmotic dehydration of apples [32]. Conversely, TPC decreased as immersion time increased (Figure 2b), implying phenol release during the immersion process. Similar trends were observed in *Himanthalia elongata* [33]. The observed inverse correlation between salt content and TPC (Figure 2c) suggests that optimal desalination conditions, with the lowest salt content, may increase the TPC.

Among the post-desalination processes, SN and VP release the internal liquids and gases of the sample, respectively, allowing distilled water to fill its spaces, including SN through high-frequency ultrasound and VP by vacuum and atmospheric pressure [34,35]. Although SN and VP exhibited comparable desalination effects, CD was the most effective. By converting gravity into centrifugal force, CD caused the loss of internal liquid and simultaneously removed salts [36,37], achieving the highest level of desalination. This finding is similar to the results reported in a study on carambola slices [38]. Based on the results, the desalination of raw CF with OICs and CD before the drying process would potentially be more economical while also enhancing both functional compound content and safety. This approach streamlines processes, when compared with desalination with dried CF, and it reduces the drying time by CD significantly.

CD-E, which had the lowest salt content, exhibited the most potent hypnotic effect, similar to DH, associated with the highest TPC (Table 9, Figure 4). A similar correlation has also been observed between the TPC and sedative effects in *Ecklonia cava* [22]. The results demonstrated that desalination removed salt, in addition to enhancing phenol-based functionality. Similarly, the desalinated *Acanthus ebracteatus* Vahl. extracts demonstrated increased anti-cancer potential [39]. However, the pentobarbital-induced sleep test has limitations with regard to confirming the sleep quality of patients with CD-E. Therefore, polysomnographic recordings should be conducted [40]. Sleep latency decreased and NREMS increased during the initial 2 h in the CD-E group (Figure 5a,b). The temporal NREMS pattern following CD-E administration showed a significant improvement but to a lesser degree than that of DH (Figure 5c). Delta activity indicates the intensity or depth of the NREMS [41,42]. CD-E (1000 mg/kg) and DH (30 mg/kg) maintained sleep quality without affecting delta activity (Figure 6c). The results indicate that the sleep-promoting effects of CD-E are similar to those of phlorotannin in *Ecklonia cava* [43] and it can be used as a natural sleep agent derived from seaweed. However, the CD-E ingestion at 1000 mg is comparable to consumption of approximately 0.17 g salt or 425 mg sodium. This exceeds the thresholds for warning labels in Chile and Sri Lanka (400 mg sodium/100 g and 1.25 g salt/100 g, respectively) [44]. Although the CD process reduces the salt content (39.8% to 0.7%) of CF significantly and enhances the functionality of its extract, it does not sufficiently decrease salt content (17.2%) in CD-E.

## 5. Conclusions

The present study confirmed that OICs and post-desalination effectively removed salt from CF and simultaneously enhanced its functionality. The OICs were determined as 42.8 °C with 1.0 h immersion. After desalination, CD was the most effective in reducing the salt content. Conversely, CD-E exhibited the highest sleep-promoting effect, which was correlated with the lowest salt content and the highest TPC. However, the CD-E salt content remains high, and further research on the desalination processes after ethanol extraction is required. Considering the increasing trend in research on seaweed-based functional foods [45], CF desalination may have added value in enhancing resource efficiency.

## Figures and Tables

**Table 1:** Experimental range and values of independent variables in the central composite design for immersion for desalination of CF.

Independent Variables	Symbol	Range and Levels				
		−1.414	−1	0	+1	+1.414
Immersion temperature (°C)	*X*_1_	20	26	40	54	60
Immersion time (h)	*X*_2_	1	2.6	6.5	10.4	12

CF: *Codium fragile*.

**Table 2:** Central composite design matrix and values of dependent variables for immersion for desalination of CF.

Run No.		Independent Variables					
		Coded Values		Uncoded Values			
		*X*_1_	*X*_2_	*X*_1_	*X*_2_	*Y*_1_	*Y*_2_
Factorial portions	1	−1	−1	26	2.6	11.8	11.2
	2	1	−1	54	2.6	11.6	10.1
	3	−1	1	26	10.4	11.3	10.3
	4	1	1	54	10.4	13.5	8.3
Axial portions	5	−1.414	0	20	6.5	12.0	10.1
	6	1.414	0	60	6.5	13.7	8.0
	7	0	−1.414	40	1.0	11.2	11.0
	8	0	1.414	40	12.0	11.9	10.0
Center points	9	0	0	40	6.5	11.0	10.3
	10	0	0	40	6.5	11.6	9.5
	11	0	0	40	6.5	11.1	10.0

*X*_1_: immersion temperature (°C); *X*_2_: immersion time (h); *Y*_1_: salt content (%); *Y*_2_: total phenolic content (TPC, mg PGE/g).

**Table 3:** Estimated coefficients of the fitted quadratic polynomial equations for dependent variables based on the *t*-statistic.

Parameters	*Y*_1_		*Y*_2_	
	Coefficient	*p*-Value	Coefficient	*p*-Value
Constant	11.233	0.001	9.933	0.001
*X*_1_	0.551	0.001	−0.759	0.002
*X*_2_	0.299	0.017	−0.514	0.009
*X*_1_ *X*_1_	0.771	0.001	−0.392	0.045
*X*_2_ *X*_2_	0.121	0.289	0.333	0.074
*X*_1_ *X*_2_	0.600	0.004	−0.225	0.256

*X*_1_: immersion temperature (°C); *X*_2_: immersion time (h); *Y*_1_: salt content (%); *Y*_2_: total phenolic content (TPC, mg PGE/g).

**Table 4:** Response surface model equations for monitoring the effects of the independent variables on the dependent variables in immersion.

Quadratic Polynomial Model Equations	*R* ^2^	*p*-Value
*Y*_1_ = 11.233 + 0.551*X*_1_ + 0.299*X*_2_ + 0.771*X*_1_^2^ + 0.121*X*_2_^2^ + 0.600*X*_1_*X*_2_	0.965	0.001
*Y*_2_ = 9.933 − 0.759*X*_1_ − 0.514*X*_2_ − 0.392*X*_1_^2^ + 0.333*X*_2_^2^ − 0.225*X*_1_*X*_2_	0.936	0.005

*X*_1_: immersion temperature (°C); *X*_2_: immersion time (h); *Y*_1_: salt content (%); *Y*_2_: total phenolic content (TPC, mg PGE/g).

**Table 5:** Analysis of variance for dependent variables.

Dependent Variables	Sources	DF	SS	MS	*F*-Value	*p*-Value
*Y*_1_	Regression					
	Linear	2	3.1386	1.5693	26.77	0.002
	Square	2	3.4247	1.7124	29.21	0.002
	Interaction	1	1.4400	1.4400	24.56	0.004
	Residual					
	Lack of fit	3	0.0864	0.0288	0.28	0.840
	Pure error	2	0.2067	0.1033		
	Total	10	8.2964			
*Y*_2_	Regression					
	Linear	2	6.7212	3.3606	27.30	0.002
	Square	2	2.1099	1.0550	8.57	0.024
	Interaction	1	0.2025	0.2025	1.65	0.256
	Residual					
	Lack of fit	3	0.2888	0.0963	0.59	0.679
	Pure error	2	0.3267	0.1633		
	Total	10	9.6491			

DF (degrees of freedom); SS (sum of squares); MS (mean square); *Y*_1_: salt content (%); *Y*_2_: total phenolic content (TPC, mg PGE/g).

**Table 6:** Response optimization for immersion for desalination of CF.

Optimal Immersion Conditions		*X*_1_ (Immersion Temperature)		*X*_2_ (Immersion Time)	
		Coded Value	Actual Value	Coded Value	Actual Value
		+0.199	42.8	−1.414	1.0
*Y*_1_	Target value Min.	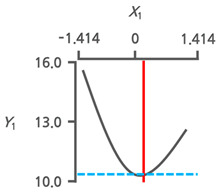		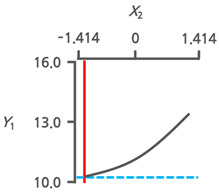	
*Y*_2_	Target value Max.	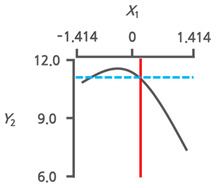		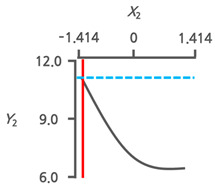	

*X*_1_: immersion temperature (°C); *X*_2_: immersion time (h). *Y*_1_: salt content (%); *Y*_2_: total phenolic content (TPC, mg PGE/g).

**Table 7:** Verification of predicted values of OICs.

	*Y*_1_	*Y*_2_
Predicted values	11.0	11.2
Experimental values	10.9 ± 0.1	11.2 ± 0.5

*Y*_1_: salt content (%); *Y*_2_: total phenolic content (TPC, mg PGE/g).

**Table 8:** Salt content and TPC of post-desalination.

Samples	Salt Content (%)	TPC (mg PGE/g)
SN	1.5 ± 0.1 ***	22.3 ± 0.2 ***
VP	1.4 ± 0.2 ***	22.6 ± 0.8 ***
CD	0.7 ± 0.2 ***	28.5 ± 0.7 ***

The data are presented as the mean ± SD (n = 3). *** *p* < 0.001, significantly different from OICs (Dunnett’s test). SN, sonication; VP, vacuum pulse; CD, centrifugal dehydration; OICs, optimal immersion conditions.

**Table 9:** Yield, salt content, and TPC of CF ethanol extracts from CF with different degrees of desalination.

Samples	Yield (%)	Salt Content (%)	TPC (mg PGE/g)
CF-E	32.0 ± 1.2	70.7 ± 0.7	7.4 ± 0.9
OICs-E	6.9 ± 0.7	60.0 ± 0.6	10.6 ± 0.1
CD-E	2.1 ± 0.1	17.2 ± 0.8	21.9 ± 0.4

Ethanol extract conditions: 70%, 60 °C, 14 h. The results were expressed as mean ± SD (n = 3). CF-E, *Codium fragile* ethanol extract; OICs-E, optimal immersion conditions ethanol extract; CD-E, centrifugal dehydration ethanol extract.

## Data Availability

The original contributions presented in the study are included in the article, and further inquiries can be directed to the corresponding authors.
